# HAS FRAILTY SCORE AND FRAILTY LETHALITY CHANGED OVER TIME? HARMONIZATION OF NHANES COHORTS FROM 1999 TO 2016

**DOI:** 10.1093/geroni/igz038.2518

**Published:** 2019-11-08

**Authors:** Joanna M Blodgett, Kenneth Rockwood, Olga Theou

**Affiliations:** 1 UCL, London, United Kingdom; 2 Dalhousie University, Halifax, Nova Scotia, Canada

## Abstract

Positive advances in life expectancy, healthcare access and medical technology have been accompanied by an increased prevalence of chronic diseases and substantial population ageing. How this impacts changes in both frailty level and subsequent mortality in recent decades are not well understood. We aimed to investigate how these factors changed over an 18-year period. Nine waves of the National Health and Nutrition Examination Survey (1999-2016) were harmonized to create a 46-item frailty index (FI) using self-reported and laboratory-based health deficits. Individuals aged 20+ were included in analyses (n=44086). Mortality was ascertained in December 2015. Weighted multilevel models estimated the effect of cohort on FI score in 10-year age-stratified groups. Cox proportional hazard models estimated if two or four-year mortality risk of frailty changed across the 1999-2012 cohorts. Mean FI score was 0.11±0.10. In the five older age groups (>40 years), later cohorts had higher frailty levels than did earlier cohorts. For example, in people aged 80+, each subsequent cohort had an estimated 0.007 (95%CI: 0.005, 0.009) higher FI score. However, in those aged 20-29, later cohorts had lower frailty [β=-0.0009 (-0.0013, -0.0005)]. Hazard ratios and cohort-frailty interactions indicated that there was no change in two or four-year lethality of FI score over time (i.e. two-year mortality: HR of 1.069 (1.055, 1.084) in 1999-2000 vs 1.061 (1.044, 1.077) in 2011-2012). Higher frailty levels in the most recent years in middle and older aged adults combined with unchanged frailty lethality suggests that the degree of frailty may continue to increase.

